# Who can you trust? A review of free online sources of “trustworthy” information about treatment effects for patients and the public

**DOI:** 10.1186/s12911-019-0772-5

**Published:** 2019-02-20

**Authors:** Andrew D. Oxman, Elizabeth J. Paulsen

**Affiliations:** 1 Centre for Informed Health Choices, Norwegian Institute of Public Health, PO Box 4404, Nydalen, N-0403 Oslo, Norway; 2 University of Oslo, Oslo, Norway

**Keywords:** Information services, Internet, Health information, Patient information, Treatments, Systematic reviews, Evidence-informed decision-making, Plain language

## Abstract

**Background:**

Information about effects of treatments based on unsystematic reviews of research evidence may be misleading. However, finding trustworthy information about the effects of treatments based on systematic reviews, which is accessible to patients and the public can be difficult. The objectives of this study were to identify and evaluate free sources of health information for patients and the public that provide information about effects of treatments based on systematic reviews.

**Methods:**

We reviewed websites that we and our colleagues knew of, searched for government sponsored health information websites, and searched for online sources of health information that provide evidence-based information. To be included in our review, a website had to be available in English, freely accessible, and intended for patients and the public. In addition, it had to have a broad scope, not limited to specific conditions or types of treatments. It had to include a description of how the information is prepared and the description had to include a statement about using systematic reviews. We compared the included websites by searching for information about the effects of eight treatments.

**Results:**

Three websites met our inclusion criteria: *Cochrane Evidence*, *Informed Health*, and *PubMed Health*. The first two websites produce content, whereas *PubMed Health* aggregated content. A fourth website that met our inclusion criteria, *CureFacts*, was under development. ***Cochrane Evidence*** provides plain language summaries of Cochrane Reviews (i.e. summaries that are intended for patients and the public). They are translated to several other languages. No information besides treatment effects is provided. ***Informed Health*** provides information about treatment effects together with other information for a wide range of topics. ***PubMed Health*** was discontinued in October 2018. It included a large number of systematic reviews of treatment effects with plain language summaries for Cochrane Reviews and some other reviews. None of the three websites included links to ongoing trials, and information about treatment effects was not reported consistently on any of the websites.

**Conclusion:**

It is possible for patients and the public to access trustworthy information about the effects of treatments using the two of the websites included in this review.

**Electronic supplementary material:**

The online version of this article (10.1186/s12911-019-0772-5) contains supplementary material, which is available to authorized users.

## Background

Patients and the public must make choices among different treatment options. We define “treatment” broadly, as any preventive, therapeutic, rehabilitative, or palliative action intended to improve the health or wellbeing of individuals or communities [1]. This includes, for example, drugs, surgery and other types of “modern medicine”; lifestyle changes, such as changes to what you eat or how you exercise; herbal remedies and other types of “traditional” or “alternative medicine”, and public health interventions. Few people would prefer that decisions about what they should and should not do for their health should be uninformed. Yet, if a decision is going to be well informed rather than misinformed, they need information that is relevant, trustworthy, and accessible. They also need to be able to distinguish between claims about the effects of treatments that are trustworthy and those that are not [2].

Often the problem is too much information rather than too little. For example, a Google search for “back pain” yields over 60 million hits [3]. PubMed, a free search engine for accessing MEDLINE and other databases maintained by the United States National Library of Medicine, includes over 27 million citations [4], and this represents only a fraction of the biomedical literature. The Cochrane Central Register of Controlled Trials, a bibliographic database that is restricted to controlled trials of treatments, contains over a million citations [5]. It is not practical for people making decisions about treatments to use search engines or databases such as these to find relevant information, critically appraise the studies they find, synthesize them, and interpret the results.

Systematic reviews reduce the risk of being misled by bias (systematic errors) and the play of chance (random errors), by using systematic and explicit methods to identify, select, and critically appraise relevant studies, and to collect and analyse data from them [6]. For information about treatment effects to be trustworthy, it should be based on systematic reviews. For it to be accessible to patients and the public, it should be easy to find and should be clearly communicated in plain language [7].

Unfortunately, a large amount of information about treatment effects is not based on systematic reviews and is not trustworthy [8–19]. This includes handouts for patients [8, 9], internet-based information [10, 11], information in social and mass media [12–18], information produced by patient organisations [8, 9, 12]. press releases [18], and advertisements [19]. Studies of the trustworthiness of health information have used a variety of criteria, but have consistently found important limitations [8–19]. Although trustworthy information about treatment effects can be found, evidence-based information is frequently written for health professionals or researchers, rather than for patients and the public [7].

There is an abundance of health information on the internet, which has become an important source of health information over the past two decades [10, 11, 20–24], but patients and the public find it difficult to search the internet for trustworthy information [21–23], and are unlikely to critically appraise the information that they do find [22, 23].

There are a number of websites that aim to improve access to trustworthy health information for patients and the public. The objectives of this study were to identify free sources of health information for patients and the public which provide information about the effects of treatments based on systematic reviews, and to evaluate those websites.

Our motivation for undertaking this review grew out of a desire to respond to people who were looking for trustworthy information about the effects of specific treatments and landed on Testing Treatments international [25], a website for promoting critical thinking about treatment claims. Rather than simply noting that the Testing Treatments website does not provide the information they were seeking, we wanted to help them by directing them to sources that do provide this information. Given this motivation, we restricted our review of websites to ones with a broad scope. There were two reasons for this. First, websites with a broad scope can meet the needs of most people seeking trustworthy information about treatment effects. Furthermore, although disease-specific websites can be useful, it would be impractical to assess any more than a small sample of websites for specific conditions or types of treatments. Second, it is easier to become familiar with one or a small number of websites than it is to use multiple websites for questions about different conditions or types of treatments.

## Methods

We considered any website that defined itself as providing “health information”, which included information about treatments. To be included in this review a website needed to be:

- Available in English
- Freely accessible (i.e. non-commercial with no cost to users or membership fees)
- That described itself as being intended for patients and the public
- Broad in scope (not limited to specific conditions or types of treatments)
- Explicitly based on systematic reviews (i.e. there had to be a description of how the information is prepared and the description had to include a statement about using systematic reviews)

We identified websites that potentially met those criteria by considering websites that we and our colleagues (see Acknowledgements) knew of. The first author (AO) searched for government sponsored websites in English speaking countries (including Australia, Canada, Ireland, New Zealand, the UK, and the USA); searched Google for “health information” and “patient information” to identify websites that are frequently accessed for health information; and checked links to other websites on the websites that were identified. On 29 January 2018, AO conducted a final set of searches using the following terms: “health information”, “patient information”, “evidence-based health information”, and “evidence-based patient information”; and these search engines: Google [3], Bing [26], DuckDuckGo [27], and HONsearch patients [28]. Google and Bing are the two most popular search engines, DuckDuckGo is not affected by your previous search history, and HONsearch searches “trustworthy” health websites. The first 20 hits for each search were screened, and any websites that looked like they might meet our inclusion criteria were checked.

AO assessed each identified website for inclusion and the second author (EP) checked those judgements using information provided on the websites. In addition, we emailed each excluded website to confirm that our reason for excluding it was correct.

AO collected the following information for each included website:

- The stated purpose
- A statement that information about treatment effects is based on systematic reviews
- Availability of links to the systematic reviews
- Reporting size of effects
- Reporting certainty of the evidence; i.e. a judgement using GRADE (Grading of Recommendations Assessment, Development and Evaluation) [29–31] or another formal approach or an informal judgement about how sure we can be about the reported effects
- Availability of links to ongoing trials
- Information about how up-to-date information about treatment effects is
- What other information is provided
- What tools there are for searching, sorting, and filtering information
- Use of plain language (i.e. summaries written for patients and the public) and the availability of a glossary

EP checked all of the information that was recorded and the judgements that were made. To inform these judgements, both authors independently searched each included website for eight questions about treatments to assess the ease of finding information (AO on 22 December 2017 and EP on 9 January 2018). We selected the eight questions by searching Google for “common health questions” and selecting the first relevant list that we found (25 Questions About Your Health Answered - Oprah.com). Many of the questions in that list were not about treatment effects and we modified some of the questions with the intention of having a variety of questions for different types of conditions and treatments. Table 1 shows the original question from that list, our question, the conditions, the treatments, and the initial search terms that we used to find information about treatment effects on each website.

**Table 1:** Questions about treatments used to assess the included websites

Original question	Our question	Condition	Treatment	Initial search terms
When should I see a doctor about......a backache?	Should I do exercises for my backache?	backache	exercise	• backache • exercise for backache • “back pain” • “back pain” AND exercise
Are the new birth control pills that eliminate your periods really safe?	Are period suppressing birth control pills safe?	birth control	period suppressing birth control pills	• period suppressing birth control pills • period suppressing “birth control pills” • “oral contraception”
Flu shots—should I or shouldn’t I?	Should I get a flu shot?	flu	flu shots	• flu shot • influenza vaccine
When should I see a doctor about...... muscle and joint pain?	Should I get my osteoarthritic knee replaced?	osteoarthritis of the knee	knee replacement	• knee replacement • surgery for osteoarthritis of the knee • osteoarthritis AND “knee replacement”
Will vitamin D save my life? Should I really be taking four times the recommended daily dose?	Should I take vitamin D to prevent osteoporosis?	osteoporosis	vitamin D	• vitamin D for osteoporosis • “vitamin D” AND osteoporosis
Will staring at a computer all day make me blind?	Should I stop using phone, tablet, computer, and TV screens before going to bed?	sleep problems	phone, tablet, computer, and TV screens	• computers and sleep problems • screens and sleep problems • computers AND insomnia • screens AND insomnia
When should I see a doctor about......a sore throat?	Should I take antibiotics for my sore throat?	sore throat	antibiotics	• sore throat • antibiotics for sore throat • “sore throat” AND antibiotics
How often do I really need to have my teeth professionally cleaned?	How often should I get dental check-ups?	tooth decay	dental checks	• dental checks • dental check-ups • routine dental check-ups

We then independently assessed what was reported about treatment effects, the consistency of reporting, and the advantages and disadvantages of each website. Disagreements were resolved by discussion. Based on these assessments and the information we had collected for each website we suggested how the websites could be improved and provided tips for website users.

For each question, we searched for information using plain language terms without Boolean logic (using the first terms shown for each question in the last column of Table 1). We recorded the number of hits for each search and each relevant summary that we found. We assessed the search as easy if we found relevant information using plain language terms without Boolean logic and the relevant information was one of the first few hits. We assessed searches as hard if we had to use technical terms or Boolean logic, or if we could not find relevant information; and as moderate if finding relevant information required some minor fiddling with the search terms or screening more than a few hits.

For each relevant summary that we found, we recorded whether any information was provided about benefits of the treatment and harms of the treatment, whether quantitative information was provided for at least one outcome, and whether a formal or informal assessment of the certainty of the evidence was provided. We then ranked the three websites for each question based on an overall assessment of how hard it was to find relevant information and the completeness of the information about the effects of the treatments.

## Results

We considered 35 websites for inclusion. Of these, 26 were excluded because information about treatment effects was not explicitly based on systematic reviews (Table 2), five were excluded because they were not intended for patients and the public (Table 3), and one was under development (Table 4). Three of the 34 websites met our inclusion criteria: *Cochrane Evidence*, *Informed Health*, and *PubMed Health* (Table 5). *Cochrane Evidence* and *Informed Health* produce content, whereas *PubMed Health*, which was discontinued in October 2018, aggregated content, including content from the first two websites.

**Table 2:** Websites excluded because they are not explicitly based on systematic reviews^a^

Website	Statements about how the information is prepared
Better Health Channel www.betterhealth.vic.gov.au	https://www.betterhealth.vic.gov.au/about/about-us "We provide health and medical information to improve the health and wellbeing of people and the communities they live in. This information is: • quality-assured and reliable • up-to-date • locally relevant • easy to understand." “We use a rigorous quality assurance and approval process to develop and review our content, including consultation and input from subject matter experts, overview by the BHC Editorial team and referral to other areas of the Victorian Department of Health as required. Our content partners are subject matter experts from a wide range of reputable Australian health, medical and academic organisations.”
Centers for Disease Control and Prevention - Diseases & Conditions www.cdc.gov/diseasesConditions/	www.cdc.gov/diseasesConditions/ Content source: Centers for Disease Control and Prevention Page maintained by: Office of Associate Director of Communication, Division of Public Affairs There are also sections on Healthy Living and Travellers’ Health. No information is provided about how the information is prepared.
Clear Health from NIH https://www.nih.gov/institutes-nih/nih-office-director/office-communications-public-liaison/clear-communication/clear-health-nih	https://www.nih.gov/institutes-nih/nih-office-director/office-communications-public-liaison/clear-communication/clear-health-nih "Clear Health from NIH Easy, accessible information and more... If you or someone close to you has received a recent diagnosis, or if you are curious because you heard about a disease, disorder, or condition on the news or from friends and want a good place to find the basic information you are looking for, this is a good place to get started …" No information is provided about how the information is prepared.
Cleveland Clinic, Health Library https://my.clevelandclinic.org/health	https://my.clevelandclinic.org/health “Access thousands of health articles, videos and tools to help manage your health” No information is provided about how the information is prepared.
Derby Teaching Hospitals, NHS, Patient information publications, http://www.derbyhospitals.nhs.uk/patients/conditions-treatments/patient-information-publications/	http://www.derbyhospitals.nhs.uk/patients/conditions-treatments/patient-information-publications/ “In this area you will be able to access our patient information publications on a range of conditions, procedures and services.” No information is provided about how the information is prepared.
Diseases https://www.canada.ca/en/public-health/services/diseases.html	https://www.canada.ca/en/public-health/services/diseases.html Government of Canada “Find information, tools and facts about symptoms, risks and how to prevent, treat and manage human diseases and illnesses.” No information is provided about how the information is prepared.
East and North Hertfordshire, NHS, Patient information leaflets http://www.enherts-tr.nhs.uk/patient-information/	http://www.enherts-tr.nhs.uk/patient-information/ “Our patient information leaflets are not meant to replace the information, advice and support provided to you by our staff but they may help answer some of your questions or help you think about questions you would like to ask your doctor or nurse” No information is provided about how the information is prepared.
Familydoctor.org https://familydoctor.org/condition/acne/	https://familydoctor.org/about/ “Familydoctor.org is the AAFP’s [American Academy of Family Physicians] award-winning consumer website, featuring physician-reviewed patient education materials, that includes care for the physical, mental, and emotional health of the whole family from newborns to older adults.” https://familydoctor.org/support-us/editorial-policy/ “Content is created by family doctors or professional writers/editors/producers who have relevant experience developing health content for patients.” "Content is reviewed by a medical review panel of family doctors to ensure that the information: • Is medically accurate, complete, and useful • Adheres to the best available evidence-based medicine, as well as AAFP policies and clinical practice guidelines • Expresses a family medicine perspective"
Health A-Z http://www.hse.ie/eng/health/az/	http://www.hse.ie/eng/health/about/ “The Health A-Z is an online database of over 600 health conditions and treatments that will support everyone living in Ireland to be well informed about their health, and that of their loved ones. The Health A-Z has been developed by the HSE’s National Clinical Programmes based on content shared by the NHS in the UK.” “NHS Choices provides open public access to a wide range of UK health information and services. NHS Choices have generously provided the baseline content used in our Health A-Z without cost to the Irish health service.”
Healthdirect www.healthdirect.gov.au	https://www.healthdirect.gov.au/about-our-content “The healthdirect team delivers comprehensive content that is clinically safe, appropriate, current and accessible, easy to understand and digest, engaging, and visually appealing.” "healthdirect editorial oversight occurs at two levels: 1. Strategic development of new services and features is managed by the Healthdirect Australia Digital Services team to ensure usability and overall quality of the website and its content. 2. Content development is overseen by the Clinical Governance team to ensure all health and clinical related content is safe, appropriate and current." “A large proportion of healthdirect’s content is comprised of links to quality information on partner websites.” “In addition to providing links to quality information on partner websites, our in-house team of health professionals, journalists and content producers create and publish our own locally-developed content. This content is reviewed for clinical accuracy every 1–4 years, depending on subject matter, and ‘Last reviewed’ dates are clearly stated on individual pages.”
Healthline https://www.healthline.com/	https://www.healthline.com/ “You can depend on us to provide expert content” https://www.healthline.com/health/about-us?ref=footer “Healthline’s medical reviewers ensure that our content is accurate, current, and user-focused. Along with extensive experience in a variety of medical specialties, they bring added perspective due to their backgrounds in clinical practice, research, and patient advocacy.”
HealthLinkBC https://www.healthlinkbc.ca/health-topics/common-health-concerns	https://www.healthlinkbc.ca/health-topics/common-health-concerns “In this section, there are information topics about some of the most common health concerns, so it is easy for you to find what you are looking for as quickly as possible.” https://www.healthlinkbc.ca/our-website “Our website provides medically-approved information on more than 5000 health and nutrition topics, symptoms, and interactive health tools and tips for maintaining a healthy lifestyle.” “The content on our website is created, reviewed, and updated by a variety of sources. This includes subject matter experts across the province, service providers, our internal clinical team, our team of registered dietitians, and our knowledgebase supplier Healthwise®.”
Health Navigator https://www.healthnavigator.org.nz/	https://www.healthnavigator.org.nz/about/quality-framework/ “we go to great lengths to ensure our website provides you with the reliable, New Zealand-focused health information you are seeking.” “The quality of information on the internet is highly variable. One of the key goals of this website is to make it easier for you to find trustworthy and reliable health information. As well as writing our own content, we link to existing resources from other reputable organisations.” http://c.ymcdn.com/sites/www.hinz.org.nz/resource/collection/0f09c2e4-7a05-49fb-8324-709f1ab2aa2f/Honey_P12.pdf?hhSearchTerms=%22Quality+and+Processes+and+Maximise+and+Health+and+Navigat%22 “Evidence bases – where possible information is based on evidence-based clinical and self management guidelines, or where these do not exist, on best or promising practice”
Mayo Clinic Patient Care and Health Information https://www.mayoclinic.org/patient-care-and-health-information	https://www.mayoclinic.org/about-this-site/product-development-policy “After the team agrees on the topic of a content piece, our writers, assisted by editorial researchers, gather best-available source materials for the topic. Best-available source materials vary by topic and may include published medical literature, evidence-based guidelines, or a Mayo Clinic physician or scientist who has distinct interest, training and expertise in the topic.” “The team follows a standardized procedure for selecting, documenting and verifying best-available medical literature, and storing references.”
MedlinePlus https://medlineplus.gov/	https://medlineplus.gov/aboutmedlineplus.html “MedlinePlus is the National Institutes of Health’s Web site for patients and their families and friends. Produced by the National Library of Medicine, the world’s largest medical library, it brings you information about diseases, conditions, and wellness issues in language you can understand. MedlinePlus offers reliable, up-to-date health information, anytime, anywhere, for free.” https://medlineplus.gov/criteria.html "MedlinePlus is designed to help you find appropriate, authoritative health information. To do this, we provide access to information produced by the National Library of Medicine and the National Institutes of Health, such as searches of MEDLINE/PubMed, our database that indexes medical research literature, and ClinicalTrials.gov, the database of clinical research studies conducted at the National Institutes of Health and many other institutions worldwide. We also provide you with a database of full-text drug and supplement information, an illustrated medical encyclopedia, a medical dictionary, and the latest health news. In addition, MedlinePlus contains pages that link to other Web sites. For example, we have Health Topic pages for over 1000 diseases and conditions from Alzheimer’s Disease to Zika Virus. We focus on organizing publications produced by the NIH Institutes and other Federal Government organizations. We also link to other Web sites, particularly ones with unique information or special features such as diagrams, glossaries, or format tailored to particular user needs."
MyHealth.Alberta.ca https://myhealth.alberta.ca/Pages/default.aspx	https://myhealth.alberta.ca/pages/About-Us.aspx “This site was built by the Alberta Gove​rnment and Alberta Health Services to give Albertans one place to go for health information they can trust. Healthcare experts across the province make sure the information is correct, up to date, and written for people who live in Alberta.” https://myhealth.alberta.ca/health/Pages/conditions.aspx?hwid=support-abouthw&%23support-abouthw-editorial “Content Written by Healthwise: Healthwise develops content through the collaborative efforts of content and medical teams using a comprehensive research and review process.” "The Healthwise® Knowledgebase contains thousands of unique references to help readers find more information on topics. Our processes and policies ensure that entry points of content give readers reasonable access to references. We include citations that: • Support statistics, particularly those that play a key role in decision making. • Support outcomes, effectiveness, or risk data. • Identify testing or treatment recommendations or guidelines. • Support prevalence data."
NHS Choices www.nhs.uk/Conditions/pages/hub.aspx	https://www.nhs.uk/aboutNHSChoices/aboutnhschoices/Aboutus/Pages/Editorialpolicy.aspx “The evidence-based knowledge that informs all NHS Choices content is derived from peer-reviewed scientific research and from the direct experience of clinicians, other health professionals, patients and the wider public. In pulling together this knowledge to provide users with a rounded and balanced package of material on a particular subject, NHS Choices requires its journalists to consult the following resources: For peer-reviewed scientific research, they consult NHS Evidence, which has developed a system for accrediting and classifying different types of research evidence with respect to its quality.”
NHS inform https://www.nhsinform.scot/	https://www.nhsinform.scot/about-nhs-inform “NHS inform is Scotland’s national health information service. Our aim is to provide the people in Scotland with accurate and relevant information to help them make informed decisions about their own health and the health of the people they care for.” https://www.nhsinform.scot/editorial-policy “All of the websites linked to from NHS inform have passed our quality assurance process. We use a range of criteria to decide if a website is suitable for inclusion, including if it’s: • free to access without a need to login or register • relevant to a Scottish audience • evidence based • updated every 12 months” “We know how important it is to create original content that reflects the healthcare needs of the Scottish population. To help us with this, we work with individuals, groups and organisations from different areas of health and social care policy and practice in Scotland to: • identify requirements • coordinate information gathering and production • provide fact checking and sign-off • agree governance arrangements”
NPS Medicinewise - Medical Info www.nps.org.au/conditions	https://www.nps.org.au/medical-info “Evidence-based resources, insights and information to improve the health of all Australians.” What we do https://www.nps.org.au/about-us#what-we-do “We provide guidance and direction on the safe and wise use of medicines and health technologies so that people stay healthier and the cost of care remains affordable. We connect and deliver meaningful information for health consumers, health professionals, government, research and other businesses to enable the best decisions about medicines, health technologies and other health choices for better health and economic outcomes. Evidence-based information is transformed into behaviour change services, digital health and data insights and knowledge transfer products.” How we do it https://www.nps.org.au/about-us#how-we-do-it “We work synergistically and in partnership with peak health organisations and government, connecting health consumers and health professionals with evidence-based resources and tools to improve the health of all Australians. We connect people with our behaviour change services, digital health and data insights and knowledge transfer products, and our work is rigorously evaluated to demonstrate impact and inform continuous improvement. We believe that well-informed health professionals and a health-savvy population are key to achieving better health and economic outcomes.”
Patient https://patient.info/	https://patient.info/about-us “Patient is the web’s leading independent health platform, established for 20 years. With more than 18 million visits a month, it is a trusted source of information for both patients and health professionals across the globe. The site contains over 4000 health information leaflets and thousands of discussion forums. It is accredited by The Information Standard, NHS England’s quality mark and was listed as ‘The top health website you can’t live without’ by The Times newspaper (Jan 2013).” “The editorial team are employed to create accurate and up-to-date content reflecting reliable research evidence, guidance and best clinical practice.”
Patient Information http://annals.org/aim/pages/patient-information	http://annals.org/aim/pages/patient-information “Annals of Internal Medicine’s “Summaries for Patients” “Summaries for Patients” are brief, non-technical summaries of studies and clinical guidelines published in Annals of Internal Medicine. The Summaries aim to explain these published articles to people who are not health care providers.” Annals of Internal Medicine’s “Patient Information Pages” ““Patient Information Pages” provide general information for the public about a common health condition. Patient Information Pages accompany each of Annals’ monthly In the Clinic feature. The pages include information about other sources for good information about the condition.”
Patient Information https://jamanetwork.com/collections/6258/patient-information	https://jamanetwork.com/collections/6258/patient-information “Explore the latest patient information from The JAMA Network, including easy-to-understand information about prevention and management of common illnesses.” No information is provided about how the information is prepared.
Penn Medicine, Patient Information, Conditions (A-Z) https://www.pennmedicine.org/for-patients-and-visitors/patient-information/conditions-treated-a-to-z	https://www.pennmedicine.org/for-patients-and-visitors/patient-information/conditions-treated-a-to-z No information is provided about how the information is prepared.
WebMD https://www.webmd.com/	https://www.webmd.com/about-webmd-policies/about-what-we-do-for-our-users “The content that we produce and the news that we feature is determined by our staff of physicians and medical journalists. It contains the latest information from reliable sources including the most important peer-reviewed medical journals, announcements from federal health agencies, and analyses on the latest health trends. Our experienced health reporters talk daily with prominent medical leaders, providing in-depth analyses, updates, and profiles that give our health news and content a perspective found nowhere else. Every original article is reviewed by our staff of full-time, board-certified physician editors.”
Wikipedia https://en.wikipedia.org/wiki/Main_Page	https://en.wikipedia.org/wiki/Health_information_on_Wikipedia “The English-language Wikipedia was estimated in 2014 to hold around 25,000 articles on health-related topics [3]. Across Wikipedia encyclopedias in all languages there were 155,000 health articles using 950,000 citations to sources and which collectively received 4.8 billion pageviews in 2013 [4]. This amount of traffic makes Wikipedia one of the most consulted health resources in the world, or perhaps the most consulted resource [4]” “A collaboration between Cochrane and Wikipedia provides access to the Cochrane Library for the purposes of incorporating their review information into Wikipedia articles.” https://en.wikipedia.org/wiki/Wikipedia:Identifying_reliable_sources#Medical_claims “Ideal sources for biomedical assertions include general or systematic reviews in reliable, third-party, published sources, such as reputable medical journals, widely recognised standard textbooks written by experts in a field, or medical guidelines and position statements from nationally or internationally reputable expert bodies. It is vital that the biomedical information in all types of articles be based on reliable, third-party, published sources and accurately reflect current medical knowledge.”

^a^These websites were excluded because they do not include a description of how information is prepared that includes a statement about using or being based on systematic reviews of research evidence. It is unclear to what extent information about treatment effects on these websites is based on systematic reviews

**Table 3:** Websites excluded because they are not intended for patients and the public^a^

Website	Statements about how the information is prepared
Epistemonikos https://www.epistemonikos.org/	https://www.epistemonikos.org/ “world’s largest systematic review database, curated and annotated by our network of collaborators.” “Articles are connected, so you can easily move from any article to all the evidence answering the same question.” “multilingual foolproof search tools” https://www.epistemonikos.org/en/about_us/who_we_are “*Epistemonikos* is aimed to health professionals, researchers and health decision-makers. It is not intended for the general public, even though it has been used by well-informed lay people and journalists successfully.”
Evidence search, NICE National Institute for Health and Care Excellence https://www.evidence.nhs.uk/	https://www.nice.org.uk/About/What-we-do/Evidence-Services/Evidence-Search “Evidence search provides access to selected and authoritative evidence in health, social care and public health.” “Sources include: British National Formulary, Clinical Knowledge Summaries, SIGN, the Cochrane Library and Royal Colleges, Social Care Online and GOV.UK.” Has filters for “Systematic Reviews” and for “Information for the Public”, but it is not possible to filter or search for information for the public that is based on systematic reviews.
PubMed https://www.ncbi.nlm.nih.gov/pubmed	https://www.ncbi.nlm.nih.gov/pubmed “PubMed comprises more than 27 million citations for biomedical literature from MEDLINE, life science journals, and online books. Citations may include links to full-text content from PubMed Central and publisher web sites.”
The Cochrane Library http://www.cochranelibrary.com/	http://www.cochranelibrary.com/about/about-the-cochrane-library.html “The Cochrane Library (ISSN 1465–1858) is a collection of six databases that contain different types of high-quality, independent evidence to inform healthcare decision-making”: Cochrane Database of Systematic Reviews, Cochrane Central Register of Controlled Trials, Cochrane Methodology Register, Database of Abstracts of Reviews of Effects, Health Technology Assessment Database, NHS Economic Evaluation Database.
Trip (Turning Research Into Practice) https://www.tripdatabase.com/	https://www.tripdatabase.com/ “Trip medical database, a smart, fast tool to find high quality clinical research evidence.” “Millions of articles items indexed & uniquely ranked” https://www.tripdatabase.com/about "Trip is a clinical search engine designed to allow users to quickly and easily find and use high-quality research evidence to support their practice and/or care. Trip has been online since 1997 and in that time has developed into the internet’s premier source of evidence-based content. Our motto is ‘Find evidence fast’ and this is something we aim to deliver for every single search. As well as research evidence we also allow clinicians to search across other content types including images, videos, patient information leaflets, educational courses and news." https://www.tripdatabase.com/about#s5 “Our most recent survey indicated that approximately 70% of our users were clinicians and 30% were non-clinical e.g. information specialists, patients or carers. Of the 70% of clinician users about 50% were doctors with an even split between primary and secondary care.”

^a^These websites were excluded because they are not primarily intended for patients and the general public. However, some patients and members of the general public use these databases

**Table 4:** Websites under development^a^

Website	Statements about how the information is prepared & status of the website
CureFacts https://www.curefacts.com/	https://www.curefacts.com/ “CureFacts provides a scientific based rating of all types of medical treatments. Our rating is mainly based on statistical summaries of all valid clinical trials. Such summaries, called Systematic Reviews, provide the highest level of evidence regarding the safety and effectiveness of treatments.” https://www.curefacts.com/about/company “We provide everyone with free access to a reliable and user-friendly information about the effectiveness and safety of medical treatments and procedures of all types: surgeries, prescription and non-prescription drugs, medical devices, vitamins, minerals, food supplements, alternative medicine and diagnostic tests.” https://www.curefacts.com/frequently-asked-questions “CureFacts relies primarily on Systematic Reviews, which are done by independent organizations and researchers, and summarize all valid clinical trials, and therefore are regarded as the most reliable type of scientific evidence. This sets us apart from other online resources, which usually rely on only one clinical trial, or on regulation guidelines that may be influenced by conflict of interest and by lobbying.” “Systematic Reviews summarize all valid clinical trials, and therefore provide the most reliable information about the effectiveness of medical treatments. However, when a treatment is introduced to the market and used by many patients, new safety issues, that were not previously detected in clinical trials (and therefore were not covered by Systematic Reviews) may arise. Therefore, we use additional sources to include known side effects and safety issues related to specific treatments. In a similar manner, we receive continuous feedback from doctors and healthcare providers, and use it to improve our database.” “We are currently running our Alpha version for which the access is limited and requires registration and then permission via email. Following further testing, we will open it to the public, and you will be able to use it without registration. You will need to register, however, if you want to keep your records (medical treatments that you want to save) and preferences (so that we can suggest treatments that people like you prefer), or if you wish to get our newsletter, [notifications] and updates.” “Currently, CureFacts has no sources of income. We are creating the first ever evidence based medicine platform, a meeting point for science, patients and doctors. In the future, we will provide you with a list of healthcare providers, doctors and on-line consultants; in turn, these doctors and consultants may pay us referral fees. This will enable us to maintain our operation, and to provide free access to all, while keeping us neutral and objective (since we will not gain anything when you choose a specific medical treatment).”

^a^Last accessed 14 February 2018

**Table 5:** Included websites

Website	*Cochrane Evidence* www.cochrane.org/evidence	*Informed Health* www.informedhealth.org	*PubMed Health* https://www.ncbi.nlm.nih.gov/pubmedhealth/
Link to information about the website	https://www.cochrane.org/what-is-cochrane-evidence ^a^ https://www.cochrane.org/about-us	https://www.informedhealth.org/informed-health.2169.en.html	https://www.ncbi.nlm.nih.gov/pubmedhealth/finding-systematic-reviews/
Stated purpose	“Cochrane Reviews are systematic reviews of primary research in human health care and health policy, and are internationally recognized as the highest standard in evidence-based health care resources. They investigate the effects of interventions for prevention, treatment, and rehabilitation.” “We are working to make this evidence accessible here on cochrane.org by creating summaries of these systematic review findings.”	“InformedHealth.org is the English-language version of the German website Gesundheitsinformation.de. By publishing this bilingual website, the Institute for Quality and Efficiency in Health Care (IQWiG, Germany) fulfils part of its legal mandate to educate the public in matters of health. The website addresses both patients and (healthy) consumers by offering a wide range of different topics.”	“PubMed Health specializes in systematic reviews of clinical effectiveness research. We include:” • Plain language summaries and abstracts of Cochrane reviews • Abstracts (short technical summaries) of systematic reviews in DARE, the Database of Abstracts of Reviews of Effects—many of them with a critical summary of the review • Full texts of reviews froma growing group of public agencies • Information developed by public agencies for consumers and clinicians that is based on systematic reviews • Methods resources about the best research and statistical techniques for systematic reviews and clinical effectiveness research “In May 2016, there were over 40,000 systematic reviews at PubMed Health.”
Based on systematic reviews Is information about the treatment effects based on systematic reviews?	**Yes**	**Yes** (“mainly”) “**Research summaries:** These are objective, brief summaries of the latest findings on a research question described in the title. They usually summarize the results of studies, for instance the results of one or (rarely) several systematic reviews or IQWiG reports. They also describe the study/studies in more detail and explain how the researchers came to their conclusions.” “We mainly use systematic reviews of studies to answer questions about the benefits and harms of medical interventions.”	**Yes**
Links to systematic reviews Are there links to the systematic reviews of treatment effects?	**Yes**	**Yes** (when used as a Source)	**Yes**
Size of effects Is information provided about the size of effects or balance between benefits and harms?	**Inconsistent** ^a^	**Yes** ^a^	**Inconsistent** ^a^
Certainty of the evidence Is information provided about the certainty of the evidence about treatment effects?	**Inconsistent** There is inconsistent use of headings and content in the summaries.	**Inconsistent** The certainty of the evidence is not reported consistently.	**Inconsistent** There is inconsistent use of headings and content in the plain language summaries and the technical summaries.
Links to ongoing trials Does the website provide links to registered/ongoing clinical trials?	**No**	**No**	**No**
Updating Isinformation provided about how up-to-date information about treatment effects is?	**Yes** Date of publication included for all summaries. Date of last search provided for some, but not all.	**Yes** Updated and Next update dates on every page. Date of publication for Sources. Date of last search not reported.	**Yes** Date of publication is available for all reviews. Date of last search provided for some, but not all.
Other information Is other information provided?	**No** The summaries include some background information, the authors’ conclusions, and links to other summaries that may be of interest.	**Yes** Symptoms, causes, outlook, diagnosis, everyday life, learn more, extras	**Yes** Three books for the general public on understanding research results, some other methods resources, Behind the Headlines, and some background information
Navigation What tools are there for searching, sorting, and filtering information?	Simple search (possible to use Boolean logic)^b^ Sort by relevance, alphabetical, or date Filter by health topics, or new and updated	Simple search (not possible to use Boolean logic)^b^ Sort by relevance or date Filter by topic areas or alphabetical	Simple search (possible to use Boolean logic)^b^ Sorted by date of publication Filters for Article types, when information was added to *PubMed Health*, Content providers, and Reviews with a quality assessment It is also possible to search using the glossary (“Health A-Z”) which includes links to summaries for consumers.
Jargon Is there a glossary or are there explanations of research and medical terms?	Plain language summaries with some variability in how well written they are. Pop-up definitions for some research and medical terms (not links to longer explanations). Glossary of terms relevant for Cochrane Reviews, not medical terms.^b^	Plain language information written for patients and the public. Hyperlinks to background information (not pop-up definitions). Glossary of “medical and scientific” terms (few research terms included).	There are no plain language summaries for most of the 40,000+ systematic reviews. Hyperlinks to information in the glossary (not pop-up definitions). Glossary of terms (“Health A-Z”) that includes both medical and research terms.
Advantages What are the main advantages of the website?	Plain language summaries of a large number of systematic reviews of treatment effects translated to several other languages.	Wide range of topics with evidence-based information about treatment effects together with other information for patients and the public.	A large number of systematic reviews of treatment effects with plain language summaries for Cochrane reviews and some additional reviews, and an extensive glossary.
Disadvantages What are the main disadvantages of the website?	Inconsistent reporting. No other information (besides treatment effects).	Inconsistent reporting and certainty of the evidence is not reported.	Inconsistent reporting. Most reviews do not have plain language summaries.
Suggestions for the website How could the website be improved?	Use consistent headings and consistently report benefits and harms (or the lack of evidence), quantitative effect estimates, and the certainty of the evidence (using GRADE [21] or a similar approach) and include links to explanations about what the grades mean. Provide information about *Cochrane Evidence* policies and how summaries are prepared on the website and make this information easier to find. Add links to other information for patients and the public or include relevant information in the plain language summaries. Add a search tool that is specific for *Cochrane Evidence* (rather than for the entire Cochrane.org website, and improve the search (including removal of OR between words as the default). Improve the glossary and make it easier to find, use pop-up definitions more consistently, and include links to longer explanations.	Use consistent headings and consistently report benefits and harms (or the lack of evidence), quantitative effect estimates, and the certainty of the evidence (using GRADE [21] or a similar approach) and include links to explanations about what the grades mean. Standard explicit reporting of when information about treatment effects is based on a systematic review and when one was not available. Include date of last search. Use pop-up definitions and include more research terms (e.g. GET-IT). Improve the search tool so that it is easier to find information. Make it possible to use Boolean logic in searches.	Make it easier to browse. Use pop-up definitions. Add links to other information for patients and the public.
Suggestions for all three websites	Provide simple instructions regarding the use of Boolean logic and the use of quotations to limit searches Include links to ongoing trials		
Tips for users How should the website be used by someone looking for information about treatment effects?	This website offers quick access to plain language summaries of over 7000 systematic reviews. Access to the full systematic reviews is free for all of the reviews in many countries and to some reviews (ones that are more than 1-year old or that have paid open access) everywhere. However, the quality of the summaries (and underlying systematic reviews) varies, and the navigation tools are rudimentary, sometimes making it hard to find information. Cochrane plain language summaries also can be found in *PubMed Health* and information about treatment effects is frequently based on Cochrane Reviews in *Informed Health*.	This website provides plain language information about the effects of treatments that is mainly based on systematic reviews together with other information for a large number of conditions. However, it can be difficult to find information. Research summaries are found for some, but not all treatments.	This website offers access to over 40,000 summaries of systematic reviews, some of which (over 8000) include plain language summaries. Access to some of the full systematic reviews is free. It also includes an extensive glossary with both medical and research terms. However, the quality of the summaries (and underlying systematic reviews) varies. There are many more reviews than in *Cochrane Evidence*, but most do not include plain language summaries.
Tips for all three websites	Limit searches by using Boolean logic (inserting AND between terms (e.g. for the condition and for the treatment) and quotation marks (to indicate that words need to be next to each other; e.g. “back pain”. Use OR between different terms for the condition or between different terms for the treatment (e.g. “knee replacement” OR surgery) to expand searches. Consider searching *Epistemonikos* if you are unable to find trustworthy information (based on a systematic review) about the effects of treatments of interest using these databases. Cochrane reviews can also be found in *Epistemonikos*, a free-access database that contains scientific summaries for over 100,000 systematic reviews (not all of treatment effects) and plain language summaries for some reviews. Most if not all of the reviews in *PubMed Health* also can be found in *Epistemonikos*, It may be easier to search than *PubMed Health* and it includes translations to several other languages. However, it is not intended for patients and the public. Consider searching for links to ongoing trials, if there is important uncertainty about the effects of relevant treatments, using the World Health Organization International Clinical Trials Registry Platform Search Portal, NHS Choices or a clinical trials registry.		

^a^The headings used were inconsistent for all three

^b^We got an error message (“A technical error has occurred. Please try again later.”) when we used AND to limit searches on *Informed Health*, and no search results when we used quotation marks (e.g. “back pain”). It was possible to use this logic on the other two websites

***Cochrane Evidence*** provides plain language summaries of over 7500 Cochrane Reviews, most of which are systematic reviews of the effects of treatments. The systematic reviews and the plain language summaries are prepared and updated by Cochrane review groups. Cochrane is a global independent network of researchers, professionals, patients, carers, and people interested in health, with over 37,000 contributors from more than 130 countries.

The plain language summaries include links to the full reviews. The full reviews are available in The Cochrane Library, which can be accessed for free in countries that have a national subscription or if the review or an update was published more than one year previously. The headings and content of the plain language summaries are inconsistent. The summaries include some background information, the authors’ conclusions, and links to other summaries that may be of interest. There is variability in the quality of the summaries. Some summaries include pop-up definitions (but not links to longer explanations) for some research and medical terms, and there is a glossary of terms relevant for Cochrane Reviews available on the Cochrane website. The summaries are translated into Chinese, Croatian, Czech, French, German, Japanese, Korean, Malay, Polish, Portuguese, Romanian, Russian, Spanish, Tamil, and Thai. The glossary is only in English.

No other information regarding treatments is provided in *Cochrane Evidence*, besides the plain language summaries of Cochrane Reviews. Cochrane website, where *Cochrane Evidence* is found has other information about the Cochrane Colaboration. Navigation tools for *Cochrane Evidence* are limited to a simple search for the entire Cochrane website. It is possible to sort findings by relevance, alphabetically, or by date of publication; and to filter the summaries by broad health topics and whether the reviews are new or updated.

***Informed Health*** is the English-language version of the German website Gesundheitsinformation.de. The website is prepared by the Institute for Quality and Efficiency in Health Care (IQWiG) in Germany. IQWiG is a professionally-independent, scientific institute established under the Health Care Reform 2004.

The *Informed Health* website provides information about treatment effects together with other information for a wide range of topics. The website includes “research summaries” for some but not all treatments. “These are objective, brief summaries of the latest findings on a research question described in the title. They usually summarize the results of studies, for instance the results of one or (rarely) several systematic reviews or IQWiG reports. They also describe the study/studies in more detail and explain how the researchers came to their conclusions.” The website states that they “mainly use systematic reviews of studies to answer questions about the benefits and harms of medical interventions.” Links to systematic reviews are provided when these are used, but the reviews may not be freely available.

All of the research summaries that we examined (Additional file 1) included quantitative information about the size of the benefits, and they included frequencies for at least one outcome, but most often only for one outcome. The certainty of the evidence is not reported. All of the information is in plain language, written for patients and the public. There are hyperlinks to background information (but not pop-up definitions). There is a glossary of “medical and scientific” terms that includes primarily medical terms and few research terms.

In addition to information about treatments, *Informed Health* includes information about symptoms, causes, outlook, diagnosis, everyday life, where to learn more, and explanations (“Extras”) of topics such as how the body works, how treatments work, and types of treatments. Navigation tools for *Informed Health* include browsing by broad topic areas, an index (A to Z list) and a simple search. Search results can be sorted by relevance, the date information on the website was created, or the date it was updated.

***PubMed Health*** specialized in systematic reviews of clinical effectiveness research. It included plain language summaries and abstracts of Cochrane Reviews; abstracts (technical summaries) of systematic reviews in the Database of Abstracts of Reviews of Effects (DARE) up to 31 March 2015; full texts of reviews from public agencies; information developed by public agencies for consumers and clinicians based on systematic reviews; and methods resources about the best research and statistical techniques for systematic reviews and clinical effectiveness research. *PubMed Health* was a service provided by the National Center for Biotechnology Information at the U.S. National Library of Medicine. It was discontinued October 31, 2018 “in an effort to consolidate similar resources and make information easier to find”. It included information from over 40,000 systematic reviews from a variety of sources, but plain language summaries were not available for most of those reviews. Links to the systematic reviews were provided, but not all of the reviews were freely available.

The reporting was inconsistent. Headings, reporting of effects, and reporting of the certainty of the evidence were inconsistent. PubMed Health had an extensive glossary (Health A – Z) and background information on drugs. Navigation tools included a simple search. Search results could be sorted by date of publication and filtered by Article types (including “Consumer information”); when information was added to *PubMed Health*, Content providers (including Cochrane and IQWiG); and Reviews with a quality assessment.

None of the three included websites includes links to ongoing trials and adverse effects are not consistently reported on any of the websites. All three include information about how up-to-date the information about treatment effects is.

*PubMed Health* was the easiest website to search, despite the large number of records that it includes. However, we had difficulties searching all three websites. We found information easily in *Cochrane Evidence* and *Informed Health* for one of the eight questions in Table 1, and for three of the questions in *PubMed Health* (Additional file 1). Conversely, it was hard to find information (or we did not find any information) for the five questions in *Cochrane Evidence*, six questions *in Informed Health*, and three questions in *PubMed Health*. It was not possible to use Boolean logic when searching *Informed Health*. This was possible on the other two websites, but none of the three provided any instructions or help for searching.

When we found information, it was consistently available about benefits, but only *Informed Health* consistently reported this information quantitatively in the plain language summaries. Quantitative information was provided in the linked scientific abstracts. None of the websites consistently reported information about harms or the certainty of the evidence, although Cochrane plain language summaries in *Cochrane Evidence* and *PubMed Health* frequently reported the certainty of the evidence. When the certainty of the evidence was reported using GRADE or another systematic approach, there was not a link to an explanation of what the grade means.

Overall we were most satisfied with *Cochrane Evidence* for 2 questions, with *Informed Health* for one question, and with *PubMed Health* for 3 of our questions. We did not find information about treatment effects on any of the three websites for two questions: “Should I stop using phone, tablet, computer, and TV screens before going to bed (for insomnia)?” and “Should I get my osteoarthritic knee replaced?” *Informed Health* provided advice for the first question (“For instance, it might help to only listen to relaxing music before going to bed and keep from talking on the phone or playing computer or mobile phone games”), but no reference to research evidence for that advice. We easily found relevant systematic reviews for both of these questions in *Epistemonikos* (Additional file 1).

## Discussion

We identified three websites for patients and the public that provide free information about treatment effects based on systematic reviews. A fourth, promising website, CureFacts, was under development (Table 4), and is still under development as of February 2019. Twenty-two other websites that provide free information for patients and the public claim to provide trustworthy, evidence-based information. However, it is not possible to know the extent to which the information they provide about treatment effects is based on systematic reviews, so is therefore less likely to be trustworthy. We considered four websites that provide access to systematic reviews, but none of these is intended for patients and the public (Table 3). Nonetheless, some people may find these useful, particularly *Epistemonikos*. It includes over 100,000 systematic reviews with the abstracts translated to Arabic, Chinese, Dutch, French, German, Italian, Portuguese, and Spanish. It is aimed for health professionals, researchers and policymakers but plain language summaries are not available for most of the reviews. Although it is not intended for patients and the public, it “has been used by well-informed lay people and journalists successfully” (Table 3).

We did not consider databases that are not free, such as Trip Pro, which includes access to over 100,000 systematic reviews; or patient information from web-based medical compendia for clinicians, such as *Best Practice*, *Dynamed*, and UptoDate. We also did not consider websites that provide information for patients and the public based on guidelines, such as the UK National Institute for Health and Care Excellence (NICE) guidance for patients; or websites that are limited to specific conditions or types of treatments.

The three websites for patients and the public that explicitly provided information about treatment effects based on systematic reviews were likely to appeal to different people and their appeal may vary depending on the question being asked. We found that we preferred each of the websites for at least one of the eight questions we used as test cases (Table 1). We found *PubMed Health* somewhat easier to search, despite the large number of records it includes, and we found both Cochrane plain language summaries and Health Information research summaries when searching *PubMed Health*. Simple instructions regarding the use of Boolean logic and the use of quotations to limit searches would help improve the ease of use for all three websites. For example, the default for *Cochrane Evidence* appears to be to insert OR between words, resulting in large numbers of irrelevant hits.

All of the websites could be improved by more consistent use of headings and consistent reporting of both benefits and (especially) harms; inclusion of quantitative information about the size of the effects; and information about the certainty of the evidence based on the use of a consistent set of criteria, such as GRADE [29–31], and links to explanations of what the grades mean. Because many systematic reviews, including Cochrane Reviews, do not consistently provide this information, plain language summaries based on systematic reviews cannot always provide this information. However, they can alert users to the absence of trustworthy information about adverse effects, when this is the case, and it is possible to provide an assessment of the certainty of the evidence even when review authors have not done this [32, 33].

All three websites provided plain language summaries of systematic reviews and all three had glossaries. However, none of the websites included both pop-up short definitions (which can be quickly accessed and read as scroll overs without having to go to another webpage) and links to longer explanations (that can be easily accessed when needed).

None of the websites included links to ongoing trials. This is something that, for example, NHS Choices does [34]. This is important because when there is important uncertainty about the effects of treatments, participating in a randomised trial may be the best option for patients [35, 36].

We are not aware of any other studies that have attempted to systematically identify and evaluate websites that provide free access to information about the effects of treatments for patients and the public which is based on systematic reviews. There are thousands of websites that provide health information and we did not systematically screen all of these. Although we believe it is unlikely that there are other websites that meet our inclusion criteria, we did not consider websites for specific conditions or types of interventions, non-English language websites, or websites that were not freely accessible. Others might want to assess these and other sources of information about treatment effects in future studies.

"The evaluation criteria that we used were based on our judgement about what information is important and what is needed to make that information accessible. For example, providing a link to the systematic review enables people to go to the source of information about treatment effects for more information, if they desire. It also makes the basis of the information clear. Information about the size of effects and the certainty of the evidence is essential for making well-informed decisions. Basic search tools are necessary to make it easy to find information on the websites, and summaries that are written in plain language for patients and the public are more likely to be understandable than abstracts written for researchers or health professionals. Consistent headings, content, and use of language make it easier for users to become familiar with the websites and to find and understand information.

Our evaluation was based in part on searching for answers for eight treatment questions (Table 1). The criteria that we used to assess what we found for each question did not require a great deal of judgement. Consequently, there were only minor disagreements in our assessments (Additional file 1), and those were easily resolved. It is uncertain how representative what we found for those questions is for what would be found for other treatment questions, but we believe they provided a fair basis for assessing the websites. Moreover, we sent full drafts of this report to people responsible for each website and their corrections did not substantially alter our assessments or conclusions.

We did not evaluate the readability of the plain language summaries and, although we described other information that each website provides, we did not evaluate whether the websites provided other information that patients and the public want or need to make informed decisions; for example, information about other treatment alternatives, costs, and people’s experiences with the treatment [37, 38]. We also did not evaluate how users of the websites experience them [39]. All of these are potential areas for future research."

## Conclusions

It is possible for patients and the public to access trustworthy information about the effects of treatments based on systematic reviews using two of the three websites included in this review. However, all three of these websites could be improved and made more useful and easier to use by consistently reporting information about the size of both the benefits and harms of treatments and the certainty of the evidence, and by making it easier to find relevant information.

Searching the three websites frequently yielded much irrelevant information. Users can limit searches by using Boolean logic - inserting AND between terms (e.g. for the condition and for the treatment) and quotation marks to indicate that words need to be next to each other; e.g. “back pain”. However, this is unlikely to be obvious to novice users. Some users may want to use sources that are not intended for patients and the public, such as *Epistemonikos*, if they are unable to find information on one of these websites. They also might want to consider searching for ongoing trials, if there is important uncertainty about the effects of relevant treatments.

There are many other websites that claim to provide evidence-based or reliable information about treatments, but it is difficult to assess the reliability of the information about treatment effects provided on those websites since they do not explicitly base that information on systematic reviews.

## Additional file

Additional file 1: Appendix review of online evidence-based patient info. Assessments of three included websites. Description of data: Search results and assessments of the information found in the three included websites for eight common health questions. (XLSX 29 kb)

## Abbreviations

AO: Andrew Oxman, the first author
EP: Elizabeth Paulsen, the second author
IQWiG: Institute for Quality and Efficiency in Health Care
